# Antineoplastic Effect of a Combined Mitotane Treatment/Ionizing Radiation in Adrenocortical Carcinoma: A Preclinical Study

**DOI:** 10.3390/cancers11111768

**Published:** 2019-11-09

**Authors:** Lidia Cerquetti, Barbara Bucci, Giulia Carpinelli, Pina Lardo, Antonella Proietti, Raffaele Saporito, Guido Rindi, Elisa Petrangeli, Vincenzo Toscano, Antonio Stigliano

**Affiliations:** 1 Endocrinology, Department of Clinical and Molecular Medicine, Sant’Andrea Hospital, Sapienza University of Rome, 00189 Rome, Italy; lidia_cerquetti@hotmail.com (L.C.); pina85la@gmail.com (P.L.); vincenzo.toscano@uniroma1.it (V.T.); 2 UOC Pathological Clinic San Pietro Hospital Fatebenefratelli, 00189 Rome, Italy; bucci.barbara@fbfrm.it (B.B.); saporito.raffaele@fbfrm.it (R.S.); 3 Department of Cellular Biology and Neuroscience, Istituto Superiore di Sanità, 00161 Rome, Italy; giulia.carpinelli@iss.it; 4 Diagnostic of Laboratory Unit, Department of Clinical and Molecular Medicine, Sant’Andrea Hospital, Sapienza University of Rome, 00189 Rome, Italy; antonella.proietti@uniroma1.it; 5 Pathology Unit, University Catholic, 00168 Rome, Italy; guido.rindi@unicatt.it; 6 CNR, Institute of Molecular Biology and Pathology, 00185 Rome, Italy; elisa.petrangeli@uniroma1.it; 7 Department of Molecular Medicine, Sapienza University of Rome, 00161 Rome, Italy

**Keywords:** adrenal cancer, mitotane, radiotherapy, adjuvant therapy, small animal magnetic resonance imaging, mismatch repair enzymes

## Abstract

Mitotane (MTT) is an adrenolytic drug used in adjuvant and advanced treatments of adrenocortical carcinoma (ACC). Ionizing radiation (IR) is also used in adrenal cancer treatment, even though its biological action remains unknown. To provide a reliable in vivo preclinical model of ACC, we used mouse xenografts bearing human ACC to test the effects of MTT and IR alone and in combination. We evaluated tumor growth inhibition by the RECIST criteria and analyzed the cell cycle by flow cytometry (FCM). In the xenograft ACC model treated with MTT/IR in combination, we observed a marked inhibition of tumor growth, with strong tumor regression (*p* < 0.0001) compared to MTT and IR given alone (*p* < 0.05). The MTT results confirm its antisteroidogenic activity (*p* < 0.05) in the xenograft ACC model, revealing its ability to render cancer cells more prone to radiotherapy treatment. In addition, to explain the biological effect of these treatments on the Mismatch Repair System (MMR), we interfered with the MSH2 gene expression in untreated and MTT/IR-treated H295R and SW13 cell lines. Moreover, we observed that upon treatment with MTT/IR to induce DNA damage, MSH2 gene inhibition in both the H295R and SW13 cell lines did not allow DNA damage repair, thus inducing cell death. In conclusion, MTT seems to have a radiosensitizing property and, when given in combination with IR, is able to promote neoplastic growth inhibition, leading to a significant reduction in tumor size due to cell death.

## 1. Introduction

Adrenocortical carcinoma (ACC) is a rare endocrine malignancy with an unfavorable prognosis. In ACC patients, the overall 5-year survival rates range from 16% to 44% in different series, with prognosis being largely dependent on the disease stage at diagnosis and success of surgery [1].

Adrenalectomy represents the treatment of choice [2], though local recurrence and metastatic disease are common during follow-up [3], supporting the need for adjuvant therapy [4]. Mitotane (MTT) is the only adrenal-specific drug approved in Europe and the United States with an ascertained efficacy in ACC [5], but its use in adjuvant therapy remains a matter of debate [6]. However, published series suggest a benefit of postoperative MTT administration in prolonging recurrence-free survival in patients with macroscopic radical ACC resection [7]. Important data derived from a multicenter study based on a retrospective analysis have reported an increase in disease-free and overall survival in MTT-treated patients compared with those from two independent untreated control groups [8]. Limited data are available with regard to the efficacy of adjuvant radiotherapy in ACC. Despite the fact that ACC has often been considered radioresistant, several series suggest the effectiveness of radiotherapy in most solid tumors [9,10,11,12]. By consent, adjuvant radiotherapy of the tumor bed might be considered in patients with incomplete surgical resection or after local recurrence [13]. In a retrospective analysis of the German ACC Registry, radiotherapy of the tumor bed in an adjuvant setting showed a significantly better 5-year recurrence-free survival, but did not affect overall or disease-free survival [14]. In an another retrospective study from the United States, radiotherapy was reported to decrease the risk of local failure by 4.7 times compared with that of surgery alone [15], and the same authors recommend the use of adjuvant radiotherapy at stage II–III disease [12].

We previously demonstrated that treatment with MTT and ionizing radiation (IR) in combination was able to inhibit H295R and SW13 cell growth by irreversible G_2_ arrest compared to the results of MTT and IR alone [16]. In another paper, we have also demonstrated that G_2_ arrest was characterized by the ability of MTT to enhance the cytotoxic effects of IR by attenuating DNA repair and interfering with the activation of the mitosis promoting factor (MPF), which is mainly regulated by the degradation of cyclin B1 in the mitotic process [17]. MMR is a highly conserved repair system that corrects mismatches arising during DNA replication and safeguards genomic integrity. Down-regulation of this system has been implicated in the engagement of cell death induced by several cytotoxic anticancer agents, including IR [18,19]. In our previous in vitro study, we reported that MTT interferes with the modulation of both MSH1 and MSH2 enzymes belonging to Mismatch Repair Systems (MMRs), which could explain the radiosensitizing properties of MTT [17]. So far, there are no mutation results in the literature concerning MMRs in ACC, except for MSH2 in a few cases, consistent with the patient’s known germline mutation causing Lynch syndrome, as well as a recognized presence of microsatellite instability [20].

The aim of the present study was to investigate the molecular mechanism of the antineoplastic effect of MTT and IR in combination in an ACC xenograft model.

## 2. Results

### 2.1. Effect of Treatment on the Volume and Morphology of Tumors in the Xenograft Model

**Figure 1:**
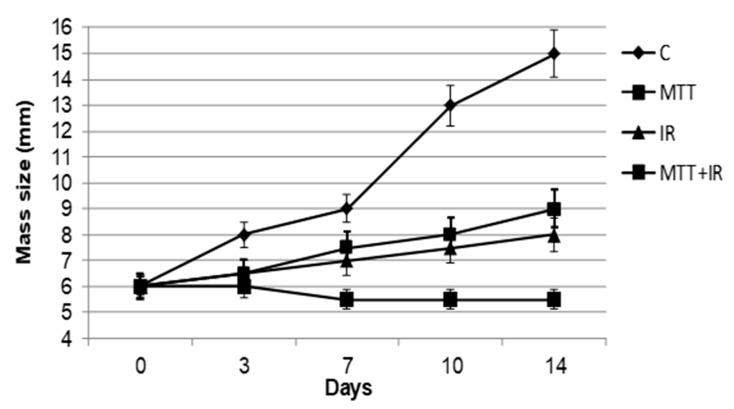
Effect of the different treatment regimens on adrenocortical carcinoma (ACC)-xenografted tumors. Tumor mass size reduction in mice receiving mitotane (MTT), ionizing radiation (IR), and MTT/IR. Both the MTT and IR treatments show a significant inhibition of neoplastic growth 7 days after the start of treatment (*p* < 0.001). Mice receiving both treatments in combination (MTT/IR) show a lasting inhibitory effect throughout the treatment (*p* < 0.0001).

The mice were stratified into five groups according to the treatment performed: six non-xenografted mice were considered as the internal negative control; six untreated xenografted mice were considered as the positive control; six xenografted mice were treated with IR; six xenografted mice were treated with MTT; and six xenografted mice were treated with MTT/IR in combination. Mice bearing tumors exposed to MTT showed a tumor mass size reduction of 20% after day 3 of treatment, showing an early ability to induce an antiproliferative effect in these tumors, which declined to 40% after 14 days (*p* < 0.001). IR treatment also induced a quick positive effect, with a mass size reduction of 20% (*p* < 0.001) after 3 days, maintaining a constant effect in the time that followed (22% after 7 days), and reaching 47% inhibition 14 days after treatment. Mice bearing tumors exposed to MTT/IR in the combined regimen showed a persistent mass regression: 25% after 3 days (*p* < 0.001), 40% after 7 days, and 63% after 14 days vs. the respective control mice (*p* < 0.0001) and significantly greater values than the results from the other groups (*p* < 0.01). The results also provide evidence of a mass size reduction at 14 days vs. 3 days of 17% in the same mice exposed to the combined regimen (Figure 1). Nevertheless, only mice receiving combined therapy exhibited persistent tumor mass regression, observing a growth proliferation trend in mice treated singularly with MTT and IR in the following days until 21 days after treatment.

### 2.2. MRI and Histological Findings Confirm the Inhibitory Effect of the Combined Treatment

**Figure 2:**
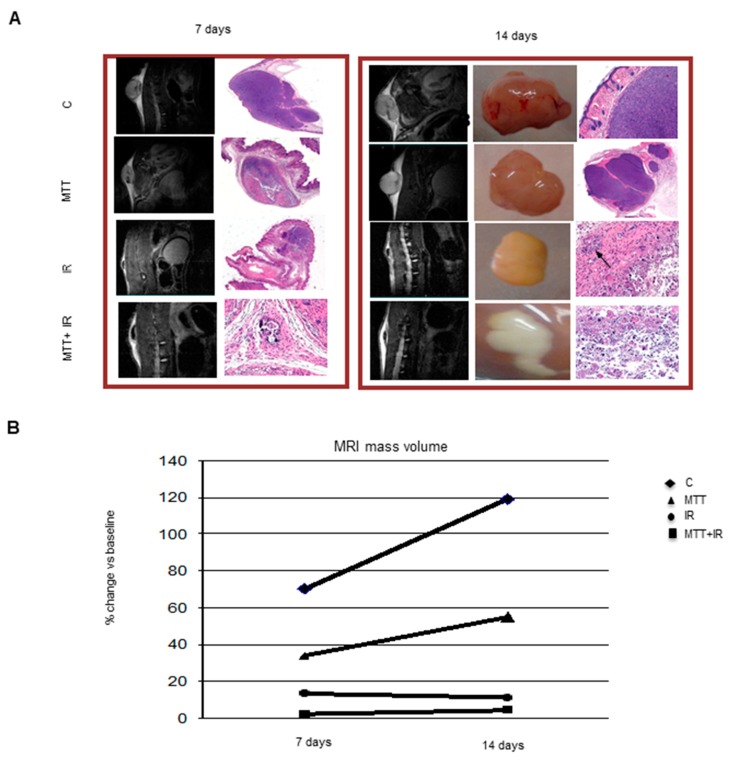
Morphological in vivo and ex vivo neoplastic appearance (**A**). Representative T2-W MRI images of tumor mass and histopathology staining at 7 and 14 days after treatment, with the corresponding macroscopic features. The images demonstrate an increase in tumor heterogeneity at the 7th day after treatment, consistent with a central area of hypointensity corresponding to tissue necrosis compared with the positive control. In particular, in mice treated with the combined MTT/IR treatment, there is evidence of a large fibrotic outline surrounding necrosis within the tumor mass. The MRI images are also consistent with the macroscopic specimens. In mice receiving the combined MTT/IR therapy, macroscopically, the tumor is replaced by a large area of fibrotic tissue at 14 days. At 14 days, slices confirm necrotic areas in the samples treated with IR and IR/MTT. The black arrows in the samples correspond to the IR treatment and show the presence of some neoplastic cellular foci. Representative graph of the tumor volume measurement by MRI (**B**). The combined IR/MTT treatment induces an early volume reduction of the tumor mass (*p* < 0.0001). A strong effect on tumor volume is evident after the IR-alone treatment as well (*p* < 0.0001). In the samples treated with MTT alone, although there is a reduction in tumor volume compared to the control (*p* < 0.001), a growth trend can be observed.

We utilized T2-weighted MRI (T2-W MRI) to monitor tumor growth. Serial imaging acquisitions were performed, starting on day 7 after the subcutaneous implantation of tumor cells and on day 14 (Figure 2A). MRI revealed a reduced growth of tumors with different treatments with respect to the control group. T2-W MRI of tumors showed differences in the volume area and was more accurate compared with caliber measurements. In particular, we observed an early volume reduction in the tumor masses treated with MTT/IR in combination compared to those from the control (*p* < 0.0001) and also a strong effect on tumors treated with IR alone (*p* < 0.0001), according to the RECIST criteria. On the contrary, in samples treated with MTT alone, though a reduction in tumor volume compared to the control was present (*p* < 0.001), we observed a growth trend 7 days after treatment (Figure 2B). Interestingly, as shown in Figure 2A, in the samples treated with MTT/IR, there appears to be evidence of an intratumoral area of necrosis.

The correspondence between MRI and the macroscopic specimen was confirmed by pathological findings. Hematoxylin and eosin slides confirmed histologic necrosis areas in samples treated with IR and MTT/IR at 14 days. Although the samples treated with IR presented some neoplastic nests, only in the samples receiving MTT/IR combined therapy did we observe macroscopic fibrosis and microscopic necrosis (Figure 2A).

### 2.3. The Treatments Induce Different Effects on Steroidogenesis

**Figure 3:**
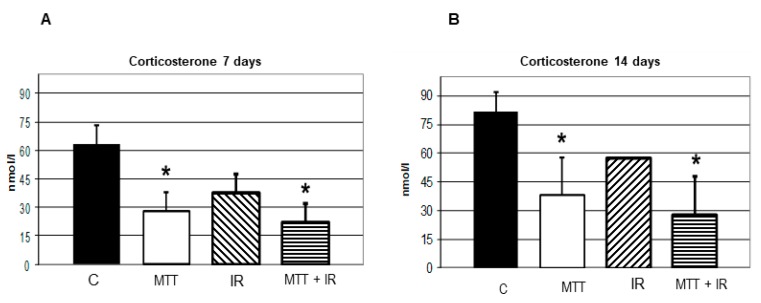
Effect of the treatments on steroidogenesis. Hormonal assays at 7 days after treatment (**A**) and at 14 days after treatment (**B**). The corticosterone levels in both groups of mice treated with the drug alone and in combination with IR are significantly lower than those from the untreated control (*p* < 0.005) at 7 and 14 days after treatment, respectively. The treatment with IR alone does not significantly affect the corticosterone level (*p* < 0.08). The combined IR/MTT treatment increases the inhibitory effect on the corticosterone level at 7 (*p* < 0.001) and 14 (*p* < 0.001) days after treatment.

MTT reduced the corticosterone level in mice after 7 and 14 days of treatment by 53% and 56%, respectively, compared to the untreated control (*p* < 0.005), confirming its antisteroidogenic properties. Contrarily, IR alone did not significantly affect steroid synthesis (*p* < 0.08). Combined MTT/IR treatment potentiated the inhibitory effect on the corticosterone level 7 (*p* < 0.001) and 14 (*p* < 0.001) days after treatment (Figure 3).

### 2.4. Combined MTT/IR Treatment Interferes with the Cell Cycle and Induces Cell Death in the ACC Xenograft Model

**Figure 4:**
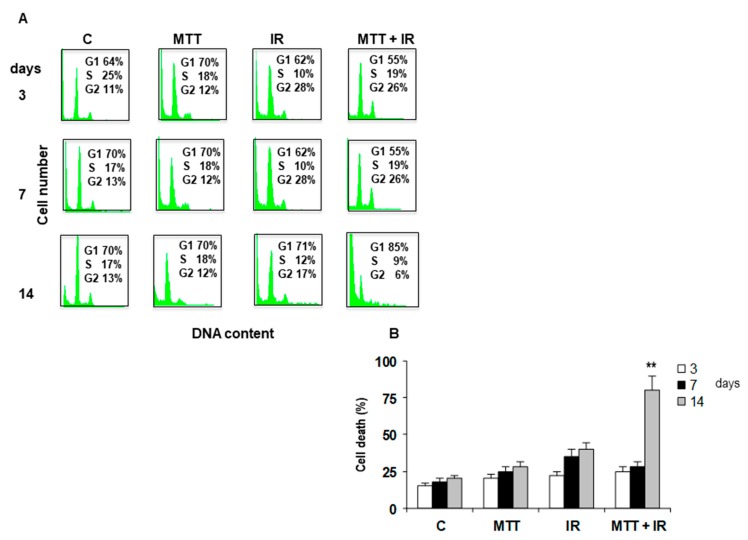
Cell cycle interference. Propidium iodide (PI) staining showing that IR induces irreversible G_2_ arrest starting from 3 to 7 days (G_2_ 28%). Conversely, IR/MTT treatment induces a decrease in the G_2_ block from 26% to 6% at 14 days (**A**). The Trypan Blue dye exclusion test shows an increase in cell death in tumor cells after 14 days from mice treated with IR/MTT compared to those from other treatments (*p* < 0.001) (**B**).

We have previously demonstrated the ability of MTT to potentiate the antiproliferative IR effect, inducing an irreversible G_2_ block of the cell cycle with an increase in cdc2 high kinase activity and an up-regulation in cyclin B1 in the ACC cell line [16]. On the basis of these data and to establish whether the antitumor effect observed in vivo was due to cell cycle modulation, a flow cytometry (FCM) analysis of tumors from untreated and treated mice was evaluated. The relative number of cells in each phase of the cell cycle was estimated from DNA Content propidium iodide (PI) staining and Cell Quest software analysis. As shown in Figure 4A, FCM analysis revealed a persistent G_2_ accumulation in tumors obtained from mice treated with IR alone at 3, 7, and 14 days. On the contrary, in MTT/IR tumor cells obtained from treated mice, a significant depletion of cells (6%) from the G_2_ phase was observed (*p* < 0.001), suggesting that the combined treatment led to irreversible DNA damage-promoted cell killing. The marked presence of cell death in the following days supported this hypothesis. Cell death was estimated by cell cycle histogram profiles, and PI staining revealed the death of 78% of cells after 14 days in the MTT/IR-treated tumors compared to those of the other treatments (*p* < 0.001) (Figure 4B).

**Figure 5:**
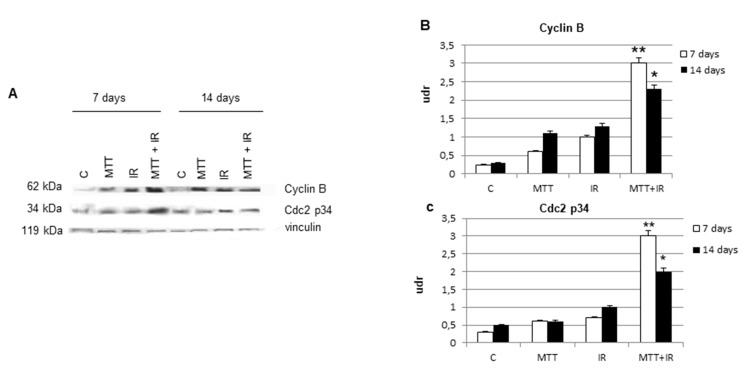
Cell cycle molecules. Western blot analysis of cyclin B and the Cdc2 p34 catalytic subunit in tumor samples from treated and untreated mice at 7 and 14 days (**A**). Bar graphs of the cyclin B and Cdc2 p34 expression levels. Cyclin B and Cdc2 p34 are significantly increased at 7 days in the MTT/IR treated samples than in other treatments (**B**,**C**), and their levels appear lower at 14 days, according to cell cycle FACS data (**B**,**C**). Densitometry readings/intensity ratio in triplicate of each band of Western blot shown in Figure 5 performed with the Image J software program. Shown in Figure S1.

To better investigate the molecular mechanisms underlying the IR G_2_ accumulation, we analyzed the expression of cyclin B and its catalytic subunit Cdc2 p34 in tumor cells obtained from treated and untreated xenograft mice. Cyclin B/Cdc2 p34 levels increased after IR exposure compared to those of untreated samples at day 7 (*p* < 0.01), indicating the stabilization of cyclin B/Cdc2 p34 due to a block in G_2_–M progression by IR treatment (Figure 5A). Consistent with previous work [16], in the MTT/IR treatment group, we observed an up-regulation of both cyclin B and its catalytic subunit (Figure 5B,C), characteristic of G_2_ arrest. However, surprisingly, the levels of both proteins were not decreased in vivo, although the cells that escaped the G_2_ phase still died.

### 2.5. Role of MSH2 in Cell Cycle Regulation

**Figure 6:**
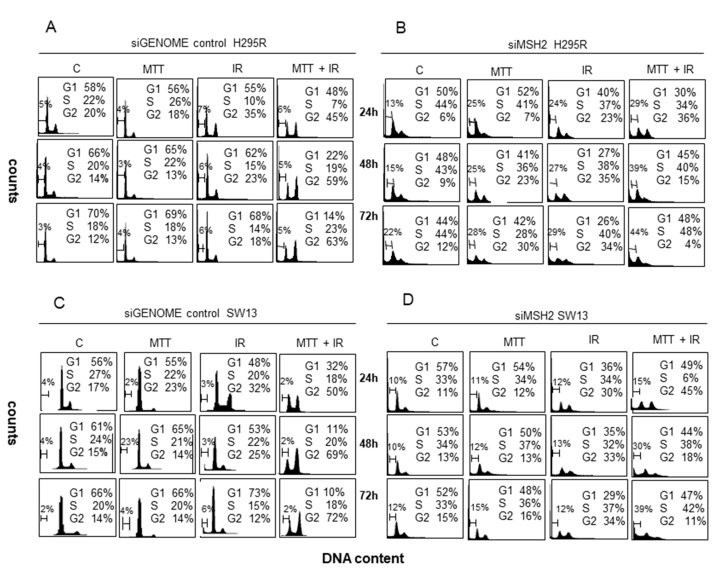
MSH2 interference in both the H295R and SW13 adrenocortical cell lines. The cell cycle was evaluated by PI staining and flow cytometry (FCM) analysis in H295R and SW13 cells transfected with siGENOME siRNA silenced with siRNA-MSH2. An irreversible block in the G_2_ phase is evident in both IR-treated siMSH2-silenced H295R and SW13 cells compared to that in siGENOME cells at 72 h (*p* < 0.001) (**A**–**D**). The combined MTT/IR treatment induces temporary G_2_ accumulation in both MSH2 interference cell lines (**B**,**D**). Cell death is already evident at 24 h in MTT/IR-treated samples in siRNA-MSH2 H295R cells (29%) (**B**). In siMSH2 H295R and SW13 cells, cell death was observed at approximately 44% and 39% at 72 h, respectively (**B**,**D**).

Previously, in ACC cell lines, we have demonstrated the ability of MTT to inhibit MLH1 and, in particular, MSH2 proteins, suggesting that it interferes with the DNA repair process [18]. To comprehend whether MTT renders cells prone to cell death after IR exposure by interfering with the MMR system, in this study, we silenced the MSH2 gene in both H295R and SW13 cell lines. As a control, we used the siGENOME siRNA, which does not target MSH2 mRNA. Western blot analysis showed that MSH2siRNA abolished MSH2 in both cell lines. As shown in Figure 6, we evaluated the cell cycle using PI staining and FCM analysis in MSH2-depleted H295R and SW13 cell lines. After IR exposure, in the siMSH2 H295R cell line, we observed accumulation in the G_2_ phase, which was more evident at 48 and 72 h after IR treatment (35% and 34%, respectively). The cell cycle modulation was associated with moderate cell death (Figure 6B). The same effect was observed in the siMSH2 SW13 cells treated with IR, in which the G_2_ arrest corresponded to 33% and 34% at 48 and 72 h, respectively. Conversely, this effect was not associated with significant cell death (Figure 6D). On the contrary, in both siMSH2 cell lines treated with MTT/IR, we observed temporary G_2_ arrest, which progressively decreased to 4% and 11% in the siMSH2 H295R and SW13 cell lines at 72 h of treatment, respectively. Therefore, the cells were not able to recover from the G_2_ arrest induced by MTT/IR treatment and were going to die, as shown in Figure 6B,D. Cell death was already increased at 24 h (29%) in siMSH2 H295R cells compared to that in siMSH2 SW13 cells (15%), from% 44 to 39%, respectively, after the MTT/IR treatment (Figure 6 B,D).

## 3. Discussion

MTT and IR in combination could represent a new strategy for fighting ACC. Our previous in vitro data on ACC cell lines have shown the capability of MTT to increase the IR effect when administered in combination [16]. In particular, IR in the H295R and SW13 cell lines induced a block in the G_2_ phase of the cell cycle, which became irreversible when MTT was added in combination. This effect was accompanied by an increase in cdc2 high kinase activity and an up-regulation of cyclin B1 [16]. In addition, we demonstrated that the cyclin B1 and cdk2 complex was strongly involved in these biological processes. In fact, the administration of purvalanol A, a selective cdk-1 inhibitor, was able to overcome cell cycle delay-induced apoptosis [17].

In the present study, we focused on the effect of this combination therapy in vivo. In particular, we assessed the effect of MTT and IR individually and in combination in an ACC xenograft model.

Treatment with MTT and IR individually was effective in inhibiting tumor growth, but a progressive resumption of tumor growth subsequently occurred, although the cytoreductive effect appeared greater for IR. Unfortunately, after ~2 weeks, the xenograft tumors showed escape from the inhibitory effect induced by IR. On the contrary, the MTT/IR combination was able to completely inhibit tumor growth. MRI was able to accurately assess the masses’ volumes during treatment. T2-W in vivo imaging allowed us to monitor therapy, demonstrating the presence of necrosis and fibrosis in the tumor samples of mice treated with both IR and MTT/IR. This feature, as shown by MRI, is supported by macroscopic observations after necroscopic examination of the animals. IR indeed was responsible for a marked reduction of the xenograft tumor only, whereas MTT/IR was not only able to reduce the tumor volume, but also showed an area of fibrotic replacement in the tumor of treated mice. Although the radiotherapy was promising, the possibility of only treating the xenograft tumors of mice with IR potentially exposes the animals to the risk of recurrence. Histological findings in the mice treated with IR indeed confirmed a significant reduction of tumor mass, even with the persistence of nests of neoplastic cells around the necrotic tissue. Interestingly, only histological samples belonging to tumors treated with MTT/IR showed the presence of necrotic tissue and the absence of neoplastic cells after 14 days of treatment. We believe this finding could provide a new avenue for the treatment of ACC.

The antisteroidogenic effect on the corticosterone level from MTT/IR treatment is significantly higher compared to that of MTT or IR alone (Figure 4). These data are consistent with our previous in vitro work [16]. Although MTT controls hormonal secretion, it alone is not capable of inhibiting cell growth. Conversely, IR itself has no effect on steroidogenesis. We believe that this combination might allow for overcoming these treatments’ singular limits, thus improving the compliance of patients affected by functioning ACC.

An analysis of tumor cell populations submitted to different treatments revealed that combined MTT/IR treatment is successful. Flow cytometry showed, in mice treated with IR alone, a consistent cellular block in the G_2_ phase until 7 days.

Surprisingly, the cells from tumors treated with MTT/IR, after a block in the G_2_ phase, underwent cell death. This is characteristic of many biological mechanisms of cell death induced by conventional protocols [21]. Therefore, we report, for the first time, the effects induced by IR on ACC in a xenograft model. We observed a constant increased level of cyclin B and the corresponding cdc2 catalytic subunit at 7 and 14 days after treatment with MTT/IR, even when cell death occurred. We speculate that the accumulation of these proteins may result from their insufficient degradation. Further experiments are needed to understand this hypothesis.

The MMR system, a family of proteins involved in DNA repair during replication, plays a role in many biological process [17,18,19]. Some studies support the idea that reducing or losing MMR function could be a promising strategy for clinical oncology [22,23]. Similarly, we observed an increased G_2_ phase block in the cell cycle and cell death in H295R and SW13 adrenocortical cell lines after silencing MSH2, an enzyme belonging to the MMR family. Combined MTT/IR treatment amplified this effect; in fact, in siMSH2, we observed G_2_ arrest at 24 h and subsequently, cells that escaped from this phase underwent cell death. Therefore, the MSH2 protein could be more involved in the cellular response to combined MTT/IR treatment through the observation of more significant cell death at all experimental time points. Furthermore, in the control sample, the absence of MSH2 protein caused cell death and a consistent accumulation of the cell proliferative compartment, indicating a pivotal role of this protein in ACC. Mimicking a genetic condition, we speculate that MTT reduces MSH2 activity, allowing IR to induce cell death.

Fassnacht et al. [14] already identified an opportunity for radiotherapy in a retrospective study in patients affected by ACC. Subsequently, others studies from Germany and the US [9,12,15] have supported these data. Sabolch et al. [12] demonstrated a significant postoperative improvement of a local control in a retrospective series of 20 patients who underwent Ro or R1 resection, followed by adjuvant radiotherapy. More recently, The European Society of Endocrinological Clinical Practice Guidelines on the management of ACC in collaboration with the European Network for the Study of Adrenal Tumors (ENSAT) suggested considering radiotherapy in selected patients (R1, RX on stage III). However, the expert panel did not reach a definitive consensus about adjuvant radiotherapy [24]. Our findings show the efficacy of radiotherapy and combination treatment with mitotane in an ACC xenograft model. These results require further confirmation in a clinical setting.

## 4. Materials and Methods

### 4.1. Cell Culture

H295R steroid-synthesizing cells were cultured in DMEM/-HAM’S F-12 medium supplemented with 50 U/mL penicillin/streptomycin and enriched with a mixture of insulin/transferrin/selenium and 10% NuSerum-I. These cells, derived from an invasive, secreting ACC, are able to establish a xenograft model in athymic mice. For injection into mice, the cells were treated with trypsin collected by centrifugation, rinsed, and resuspended in 1X phosphate-buffered saline (PBS).

The SW13 cell line was grown in Leibovitz’s L-15 medium supplemented with 10% bovine serum.

### 4.2. Animals

Four-week-old nu/nu-Forkhead female mice (Charles-River, Calco (LC), Italy) were maintained in groups of five or less and quarantined for 2 weeks. The mice were housed under controlled temperature (25 °C) and light conditions (light/dark 12 h regimen) and were allowed access to food and water ad libitum. The mice were then randomly assigned to three groups for respective treatment: xenografted mice treated with IR alone, MTT alone, and MTT/IR in combination. Additionally, two untreated control groups were used: xenografted mice alone (positive control) and non-xenografted, PBS-inoculated mice (negative control).

The tumors were obtained by the subcutaneous injection of 25 × 10^6^ H295R cells into the flanks of the mice. H295R-xenografted tumors showed characteristics and histological features similar to those of the original tumor, constituting a useful in vivo ACC model [25]. The animals were weighed daily and monitored until the tumor masses reached a volume of ~61.2 mm^3^, as evaluated by MRI imaging, which occurred 7 days after cell inoculation. Tumor growth was measured and monitored on a regular interval (twice a week), and tumor volume was calculated by assuming a prolate spheroid shape, as previously described [26].

For each group, the animals were sacrificed at 3, 7, and 14 days after beginning treatment. Drug tolerability was assessed in tumor-bearing mice in the following terms: (1) lethal toxicity, and (2) body weight loss percentage [(body weight on day x/body weight on day 1) × 100], where x represents a day after or during the treatment period [27,28]. Blood for corticosteronemia assays was collected by drawing it from the heart, separating the serum. All animal procedures and this study were approved by the local Ethics Committee for Animal Research by the Sapienza and Catholic University of Rome, Project no. 52176; Approval Date, 06 December 2011 in agreement with the Italian Ministry of Health.

### 4.3. Mitotane

MTT (Sigma-Aldrich, Milan, Italy) was resuspended in dimethyl sulfoxide (DMSO) and administered at a final volume of 20 μL/day per animal via intraperitoneal injection (0.150 mg/kg). Negative control mice were treated with the same volume of the vehicle alone (DMSO).

### 4.4. Ionizing Radiation

The groups of mice undergoing IR and IR/MTT combined treatment received a dose of 6 Gy 7 and 14 days after tumor development (6 min and 50 sec for a mouse, corresponding to the dose radiation administered at a velocity of 0.879 Gy/min) through exposure to ^137^Cs by a Gammacell 40 Caesium-137 source Research Irradiator (MDS Nordion) once, as previously described [29].

Preliminary experiments were performed to determine the correct dose rate corresponding to 6 Gy.

The H295R and SW13 cell irradiation was based on single irradiation doses of 4 Gy/min and analyzed from 24 to 72 h. All experiments were repeated at least three times, and each experimental sample was seeded in triplicate.

### 4.5. In vivo Magnetic Resonance Imaging (MRI) Study

In vivo MRI measurements were performed using a 4.7 T-horizontal-bore Varian Inova SIS 200/183 system (Varian, Palo Alto, CA, USA) equipped with a transmit volume coil in combination with an electronically decoupled receive-only surface coil (Rapid-Biomedical, Rimpar, Germany) suitable for small animals.

To assess the effect of the treatments on tumor growth, longitudinal monitoring was performed using proton MRI. Each animal was scanned every 4 days after beginning treatment. T2-weighted (T2-W) imaging was obtained by a spin echo sequence with an echo time of 50 ms, repetition time of 2500 ms, field of view of 20 × 20 mm^2^, matrix dimensions of 128 × 128 (in plane resolution of 156 μm × 156 μm), average number of 2, and slice thickness of 0.7 mm. Tumor volumes were measured by means of Varian dedicated software.

For MRI examinations, the animals were anesthetized with 1%–2% isoflurane in a mixture of 1 L/min O_2_ (Forane, Abbott SpA, Latina, Italy).

### 4.6. Histopathology

After the mice were sacrificed, the tumor tissues were removed, fixed with 10% formaldehyde, dehydrated, embedded in paraffin, and cut into sections (3–4 μm) using a microtome. The sections were then subjected to hematoxylin-eosin staining according to the standard protocol and were observed with light microscopy.

### 4.7. Tumor Cells and Cell Cycle Analysis

A single cell suspension was obtained through the homogenization of dissected tumors. The cell cycle analysis was performed by flow cytometry (FCM) after propidium iodide (PI) staining. To perform PI staining, the cells were stained with a solution containing 50 μg/mL PI (Sigma-Aldrich, Milan, Italy) and 75 KU/Ml RNase (Sigma-Aldrich, Milan, Italy). For both experiments, 20,000 events/samples were acquired using a FACScalibur cytofluorimeter (Becton-Dickinson, Sunnyvale, CA, USA).

Cell death was evaluated by the Trypan Blue dye exclusion test.

### 4.8. Steroid Assays

Blood was collected by puncturing the left heart ventricle. Serum corticosterone levels were determined in treated and untreated mice by the ELISA kit (ALPCO, Salem, NH, USA).

### 4.9. MSH2 Silencing

To perform transient transfection experiments, 3.5 × 10^5^ H295R and SW13 adrenocortical cells were grown in complete medium and, 24 h after seeding, were transfected by Lipofectamine with a 200 nM final concentration of siRNA-MSH2 oligonucleotides (Dharmacon, Lafayette, CO, USA). The cells were incubated for 12 h, according to the manufacturer’s instructions. After overnight incubation, the cells were maintained in fresh medium and then exposed to IR, MTT, and IR/MTT.

Cells were transfected with a negative control siGENOME by non-targeting siRNA MSH2 (Dharmacon, Lafayette, CO, USA) by Fugene HD (Promega, Madison, WI, USA), according to the manufacturer’s protocol.

### 4.10. Western Blotting Analysis

Total protein (50 μg) was electrophoresed on a polyacrylamide gel with SDS and transferred onto a nitrocellulose membrane. Treated and untreated cells were incubated with anti-cyclin B1 1:200, cdc2 p34 1:200, and anti-MSH2 1:250 (Santa-Cruz Biotechnology, CA, USA) and Vinculin 1:10,000 (Sigma-Aldrich, St. Louis, MO, USA). Band analysis was performed with the Image J (Image Processing and Analysis in Java) software program.

### 4.11. Statistical Analysis

All experiments were repeated at least three times, and each experiment was carried out at least in duplicate. The data are presented as means ± standard deviation (SD). A comparison of the individual treatments was conducted using Student’s *t* test employing at least three independent data points. A *p*-value < 0.05 was considered significant. Image J software was used.

## 5. Conclusions

In summary, we believe that the results emerging from the literature regarding the potential of radiotherapy in ACC treatment, in combination with those produced in this work, demonstrate important requirements for the development of future clinical strategies to counteract ACC.

## Supplementary Materials

The following are available online at https://www.mdpi.com/2072-6694/11/11/1768/s1, Figure S1: Densitometry readings/intensity ratio in triplicate of each band of Western blot shown in Figure 5 performed with the Image J software program.
