# Supplementary material for: Antineoplastic Effect of a Combined Mitotane Treatment/Ionizing Radiation in Adrenocortical Carcinoma: A Preclinical Study
Source: Cancers (Basel). 2019 Nov 9;11(11):1768. doi: 10.3390/cancers11111768 (PMC6895792; doi:10.3390/cancers11111768)
Supplement: Supplementary file 1 [file cancers-11-01768-s001.pdf]

Antineoplastic Effect of a Combined Mitotane Treatment/Ionizing Radiation in Adrenocortical Carcinoma: A Preclinical Study

Lidia Cerquetti, Barbara Bucci, Giulia Carpinelli, Pina Lardo, Antonella Proietti, Raffaele Saporito, Guido Rindi, Elisa Petrangeli, Vincenzo Toscano and Antonio Stigliano

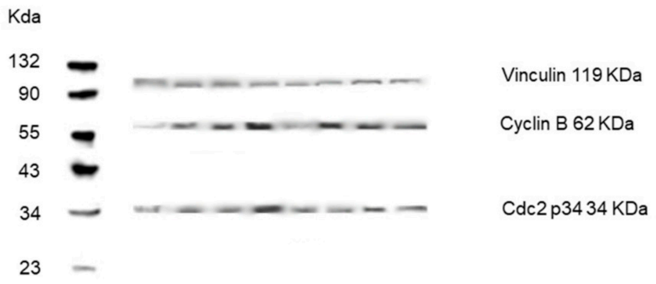

**Cyclin B 7 days**	
C	3.052.246
MTT	5.337.569
IR	7.045.841
MTT+IR	13.852.196
**Cyclin B 14 days**	
C	972.891
MTT	6385,761
IR	9.697.154
MTT+IR	12.324.857

**Cdc2 p34 7 days**	
C	5.686.167
MTT	4.536.205
IR	5.567.418
MTT+IR	13.910.468
**Cdc2 p34 14 days**	
C	2.013.125
MTT	3.093.962
IR	6.794.205
MTT+IR	11.010.619

**vinculin 7 days**	
C	12.516.894
MTT	6.954.205
IR	7.952.004
MTT+IR	4.665.640
**vinculin 14 days**	
C	3.530.083
MTT	5.151.104
IR	7.031.790
MTT+IR	5.651.320

Figure S1. Densitometry readings/intensity ratio in triplicate of each band of Western blot shown in Figure 5 performed with the Image J software program.

© 2019 by the authors. Licensee MDPI, Basel, Switzerland. This article is an open access article distributed under the terms and conditions of the Creative Commons Attribution (CC BY) license (http://creativecommons.org/licenses/by/4.0/).
